# RTA-408 attenuates the hepatic ischemia reperfusion injury in mice possibly by activating the Nrf2/HO-1 signaling pathway

**DOI:** 10.1515/biol-2025-1093

**Published:** 2025-04-24

**Authors:** Huilian Hua, Yao Quan, Zhiqin Li, Bo Pan, Fang Zhang, Jun Wang, Jindong Li, Su Jiang

**Affiliations:** The Affiliated Taizhou People’s Hospital of Nanjing Medical University, Taizhou School of Clinical Medicine, Nanjing Medical University, Taizhou, Jiangsu, 225300, China; Department of Pharmacy, The Hospital Affiliated to Medical School of Yangzhou University (Taizhou People’s Hospital), Taizhou, Jiangsu, 225300, China

**Keywords:** RTA-408, Nrf2, HO-1, oxidative stress, hepatic ischemia reperfusion injury

## Abstract

RTA-408, also referred to as Omaveloxolone, is a potent activator of nuclear factor erythroid 2-related factor 2 (Nrf2) and has been demonstrated with protective effects against oxidative stress-induced injury. Oxidative stress is closely associated with the pathogenesis of hepatic ischemia reperfusion injury (HIRI). The aim of this study is to elucidate the impact and underlying mechanisms of RTA-408 in the process of HIRI. In the HIRI mice models, we found that RTA-408 improved liver function of HIRI mice and attenuated the HIRI-induced oxidative stress *in vivo*. Moreover, the neutrophil infiltration in liver tissues of HIRI mice was alleviated by the administration of RTA-408. RTA-408 treatment also rescued the elevated apoptosis in the liver tissues of HIRI mice. Furthermore, we demonstrated that RTA-408 treatment activated the Nrf2/HO-1 signaling pathway in liver tissues of HIRI mice. Furthermore, the HIRI mice models were developed using Nrf2-deficient mice to explore whether the protective effect of RTA-408 on HIRI was achieved through the activation of Nrf2. The results indicated that RTA-408 did not significantly alleviate the liver injury in Nrf2-deficient mice. Collectively, our results suggest that RTA-408 attenuates HIRI by improving liver function, and attenuating oxidative stress damage, apoptosis and inflammatory response possibly via the Nrf2/HO-1 pathway, which may provide a novel treatment strategy for HIRI patients.

## Graphical abstract

**Figure:**
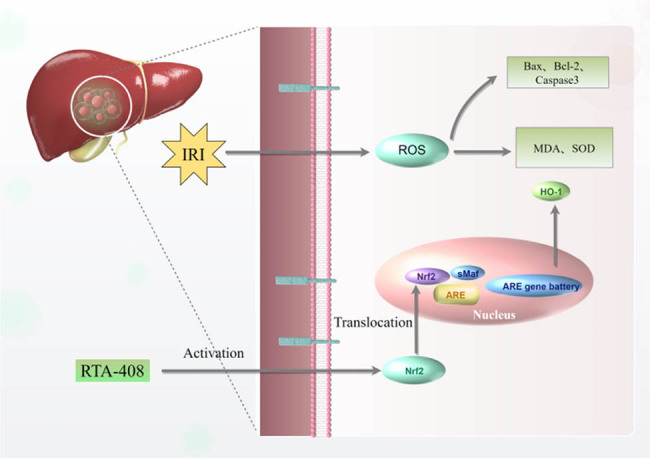

## 1 Introduction

Hepatic ischemia reperfusion injury (HIRI) is a severe and inevitable complication of some liver surgeries such as hepatectomy and liver transplantation due to the high dependence of liver on oxygen supply, which significantly affects the survival of patients [1,2]. HIRI is characterized by the initial blockade of blood flow to the liver, followed by the restoration of perfusion along with reoxygenation, which usually induces tissue damage and inflammatory responses and leads to liver dysfunction or failure [3]. The mechanisms involved in HIRI are complicated, including oxidative stress, cell death, inflammatory responses, and others [4].

For example, tumor necrosis factor (TNF) activates the extracellular signal-related kinase, c-Jun NH2-terminal kinase (JNK), and NF-B, which are potent inducers of inflammation. In addition to its proinflammatory role, TNF is an anti-tumor cytokine and was reported to activate NF-B formation [5]. Besides, there is a pathway, which can also lead to widespread protein destabilization and DNA damage through redox instability and shifts in energy homeostasis [6]. The status of liver function is often identified by assessing the alanine transaminase (ALT) and aspartate transaminase (AST) levels in blood serum. In instances where hepatocytes are subjected to damage, these enzymes leak into circulation and are consequently markers for liver damage [7].

Currently, it remains challenging to manage HIRI in clinical practice, and understanding its underlying mechanism is essential for the exploration of effective treatment methods.

Omaveloxolone (RTA-408) is a new synthetic oleanane triterpenoid compound, which is known as a powerful activator of Nrf2. Previous literature has revealed the anti-oxidant and anti-inflammatory properties of RTA-408 in clinical trials. For example, RTA-408 is demonstrated to be safe and effective on improving the neurological function in patients with Friedreich ataxia [5]. RTA-408 is also suggested to be safe and well tolerated in patients with advanced solid tumors and can activate the Nrf2 signaling in a dose-dependent way [8]. Moreover, multiple studies have demonstrated the cytoprotective effects of RTA-408 after injury. It is indicated that RTA-408 enhances microglial polarization by suppressing reactive oxygen species (ROS) production and activating Nrf2 in mice after intracerebral hemorrhage [9]. In addition, RTA-408 can attenuate the oxidative stress-induced damage in human retinal pigment epithelial cells via activating Nrf2 [10]. In our previous study, RTA-408 is found to improve the renal function, reduce ROS generation, and down-regulate the oxidative injury marker in mice with ischemia-reperfusion injury by activating Nrf2 and up-regulating the GSH biosynthetic enzyme [11]. Furthermore, RTA-408 is suggested with hepatoprotective functions in mice with nonalcoholic steatohepatitis, which are achieved by reducing inflammatory responses and fibrosis through Nrf2 [12]. However, the impact of RTA-408 on HIRI is rarely reported, which requires further investigations.

Nrf2 encodes a transcription factor that belongs to the basic leucine zipper (bZIP) family of proteins and regulates genes containing the antioxidant response element and encoding proteins implicated in the response to injury and inflammation [11]. Accumulating evidence has defined the cytoprotective role of Nrf2 in ischemic-reperfusion injuries, including HIRI, by reducing the oxidant stress damage and inflammatory responses [13,14,15]. Moreover, Nrf2-deficient mice are reported with enhanced inflammatory responses, higher ROS levels and severe injury in the HIRI mice models [16,17,18], suggesting the hepatoprotective functions of Nrf2 in liver injury.

The aim of the present study was to elucidate the impact and underlying mechanisms of RTA-408 in HIRI. We hypothesized that RTA-408 activated Nrf2 signaling to alleviate oxidative stress, mitigate inflammation, and improve liver function in HIRI mice. The findings of our work may provide theoretical basis for the application of RTA-408 in treating HIRI.

## 2 Materials and methods

### 2.1 Animal experiment

Male wild-type C57BL/6J mice (8 weeks, 20–25 g) and *Nrf2-*deficient mice (*Nrf2*−/−mice; 8 weeks, 20–23 g) were obtained from Jiangsu Huachuang Xinnuo pharmaceutical Technology Company. Mice were housed at 24 ± 2°C in a 12/12 h light/dark cycle with free access to food and water. Wild-type mice were randomized into the sham, HIRI, and HIRI + RTA-408 groups (*n* = 6 per group). *Nrf2*−/− mice were also allocated into the HIRI and HIRI + RTA-408 groups (*n* = 3 per group). HIRI mice models were generated as previously described [19,20]. One day before the hepatic ischemia, mice in the HIRI + RTA-408 group were intraperitoneally injected with 100 μg/kg RTA-408 (MedChemExpress, USA). Mice were anesthetized by intraperitoneal injection of Tribromoethanol (0.2 mL/10 g), followed by laparotomy. The arterial/portal vessels to the cephalad lobes were clamped for 60 min. Then, the clamps were removed for reperfusion for 24 h. Mice in the sham group received the same operation with no occlusion of vessels. Next mice were euthanized and the liver and blood samples were obtained.


**Ethical approval:** The research related to animal use has been complied with all the relevant national regulations and institutional policies for the care and use of animals, and has been approved by the Ethics Committee of Jiangsu Huachuang Xinnuo Pharmaceutical Technology Company.

### 2.2 ALT and AST measurement

Mice blood samples were collected from the eye socket and centrifuged for 20 min at 1,000 × *g*. Serum ALT and AST levels were determined using an ALT kit (ml076532, Shanghai Enzyme-linked Biotechnology Co., Ltd) and AST kit (ml077324, Shanghai Enzyme-linked Biotechnology Co., Ltd) in accordance with the manufacturer’s instructions.

### 2.3 Hematoxylin and eosin (H&E) staining

The collected mice liver samples were processed with 4% formaldehyde for 24 h, dehydrated, paraffin-embedded, and sliced into sections (4 μm in thickness). Next samples were subject to H&E staining (C0105, Beyotime, Shanghai, China). The histological changes were observed using an OLYMPUS microscope (CKX53). Hepatic injury score was evaluated using the Suzuki’s method [13].

### 2.4 Immunohistochemistry (IHC)

Primary MPO antibody (ab208670, 1:1,000, abcam) was cultured with mice liver sections overnight at 4°C. Subsequently, the sections were cocultured with the corresponding secondary antibody (ab205718, 1:2,000, abcam) for 60 min at ambient temperature. After washing with tris-buffered saline with Tween-20 (TBST) thrice, a DAB kit (ZLI-9018, Zsbio, China) was used for color development. Finally, the percentage of MPO-positive was analyzed by Image J.

### 2.5 TdT-mediated dUTP nick-end labeling (TUNEL)

Cell apoptosis in mice liver samples was assessed using an *in situ* cell death detection kit (HY-K1091, MCE) in accordance with the manufacturer’s protocol. The tissues were dehydrated, incubated with proteinase K, and cultured with TUNEL solution, followed by counterstaining hematoxylin. An OLYMPUS microscope was applied to capture the images in each group. The percentage of TUNEL-positive was analyzed by Image J.

### 2.6 Western blot

Cytoplasmic and nuclear protein were extracted with a cytoplasmic/nuclear protein enrichment kit (P0027, Biyun Tian, China) following the manufacturer’s protocol. Mice liver samples in each group were treated with lysate buffer (P0013C, Biyun Tian). Then, the protein samples were separated by gel electrophoresis, followed by electro-transfer to the nitrocellulose membranes (Sigma-Aldrich) blocked by 5% nonfat milk, followed by coincubation with anti-Bax (ab182733, 1:2,000, abcam), anti-Bcl-2 (ab182858, 1:2,000, abcam), anti-cleaved caspase-3 (ab214430, 1:5,000, abcam), anti-Nrf2 (A21176,1:1,000, Abclone), anti-Histone H3 (HY-P80166, 1:1,000, MCE), and anti-HO-1 (ab52947, 1:2,000, abcam) overnight at 4°C, with β-actin as the internal control. After rinsing with TBST thrice, the membranes were cultured with the secondary antibodies at ambient temperature for 60 min. Finally, the protein bands were visualized with an enhanced chemiluminescence kit and ImageJ software was applied for protein quantification.

### 2.7 Malondialdehyde (MDA) and total superoxide dismutase (SOD) measurement

The MDA content and SOD activities were determined using a lipid peroxidation. MDA and SOD levels were measured using the respective assay kits (MDA: A003-1-2, SOD: A003-3-2, Nanjing Jiancheng, China) according to the manufacturer’s protocols.

### 2.8 Statistical analysis

GraphPad Prism 8.0 software was applied for data analysis. Data are exhibited as mean value ± standard deviation. The statistical significance of multiple-group difference was assessed by one-way analysis of variance. *P* < 0.05 indicated statistical significance.

## 3 Results

### 3.1 RTA-408 attenuates liver injury in HIRI mice

**Figure 1:**
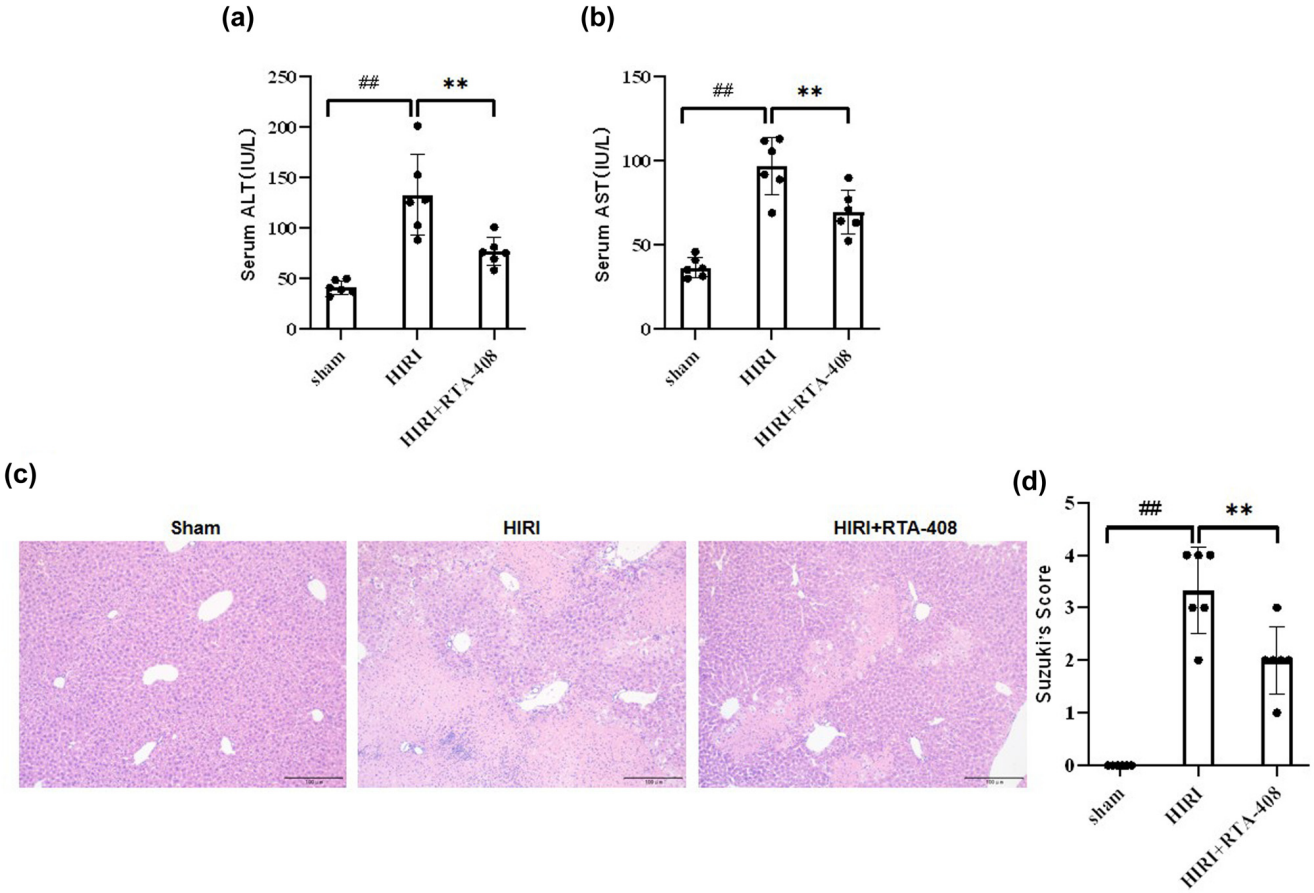
RTA-408 alleviates liver injury and improves liver function in HIRI mice. Serum (a) and (b) ALT and AST levels in each group, *n* = 6. (c) and (d) H&E staining and Suzuki’s score for the morphology examination of mice liver samples, *n* = 3, (×200). ^##^ *P* < 0.01 vs Sham group, **P < 0.01 vs HIRI group.

HIRI mice models were generated to elucidate the effects of RTA-408 *in vivo*. We found that the serum ALT and AST levels increased in the HIRI group were significantly reduced by the administration of RTA-408 (Figure 1a and b). The results of H&E staining showed that mice liver tissues in the HIRI group exhibited significant damage, lobular structure collapses, necrosis, and inflammatory cell infiltration, and the treatment of RTA-408 was revealed to attenuate the liver injury in HIRI close to normal morphology, with the significantly reduced Suzuki’s score in comparison with the HIRI group (Figure 1c and d). Overall, these results indicated that RTA-408 alleviated the liver injury and improved the liver function of HIRI mice.

### 3.2 RTA-408 mitigated the inflammatory response in HIRI mice

**Figure 2:**
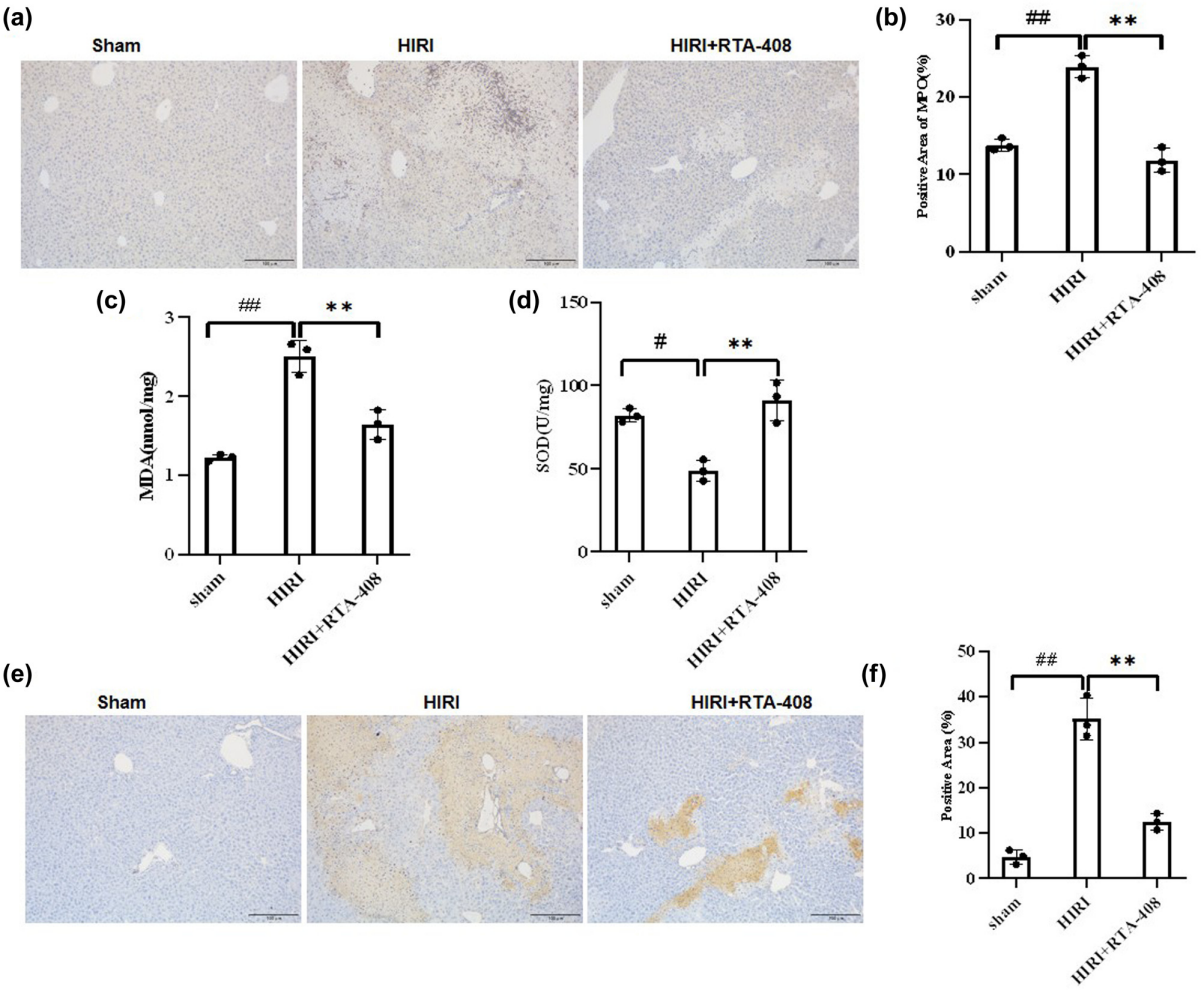
RTA-408 reduces the HIRI-induced inflammatory reaction, cell apoptosis, and oxidative stress in mice. (a) and (b) The ratio of MPO-positive in mice hepatic tissues were evaluated by IHC, (×200). (c) and (d) MDA content and SOD activities in mice hepatic tissues. (e) and (f) TUNEL staining was conducted to assess the hepatic apoptosis of mice in indicated groups, *n* = 3, (×200). ^##^ *P* < 0.01and ^#^ *P* < 0.05 vs Sham group, **P < 0.01 vs HIRI group.

The impact of RTA-408 on HIRI-induced inflammatory response was explored by detecting the level of proinflammatory factors in mice liver tissues. The neutrophil infiltration in mice hepatic tissues was then evaluated. The results of IHC reveled that elevation in the number of MPO-positive cells in HIRI group was counteracted by RTA-408 administration (Figure 2a and b).

### 3.3 RTA-408 repressed the oxidative stress induced by HIRI *in vivo*

The effects of RTA-408 on oxidative stress in HIRI mice models were investigated. The MDA content in mice hepatic tissues showed significant elevation while the SOD activities exhibited significant reduction in HIRI mice. The administration of RTA-408 was demonstrated to reverse the HIRI induced changes in oxidative stress markers in the HIRI + RTA-408 group (Figure 2c and d). Collectively, RTA-408 alleviated the HIRI-induced oxidative stress *in vivo*.

### 3.4 RTA-408 reduced the liver cell apoptosis in HIRI mice

**Figure 3:**
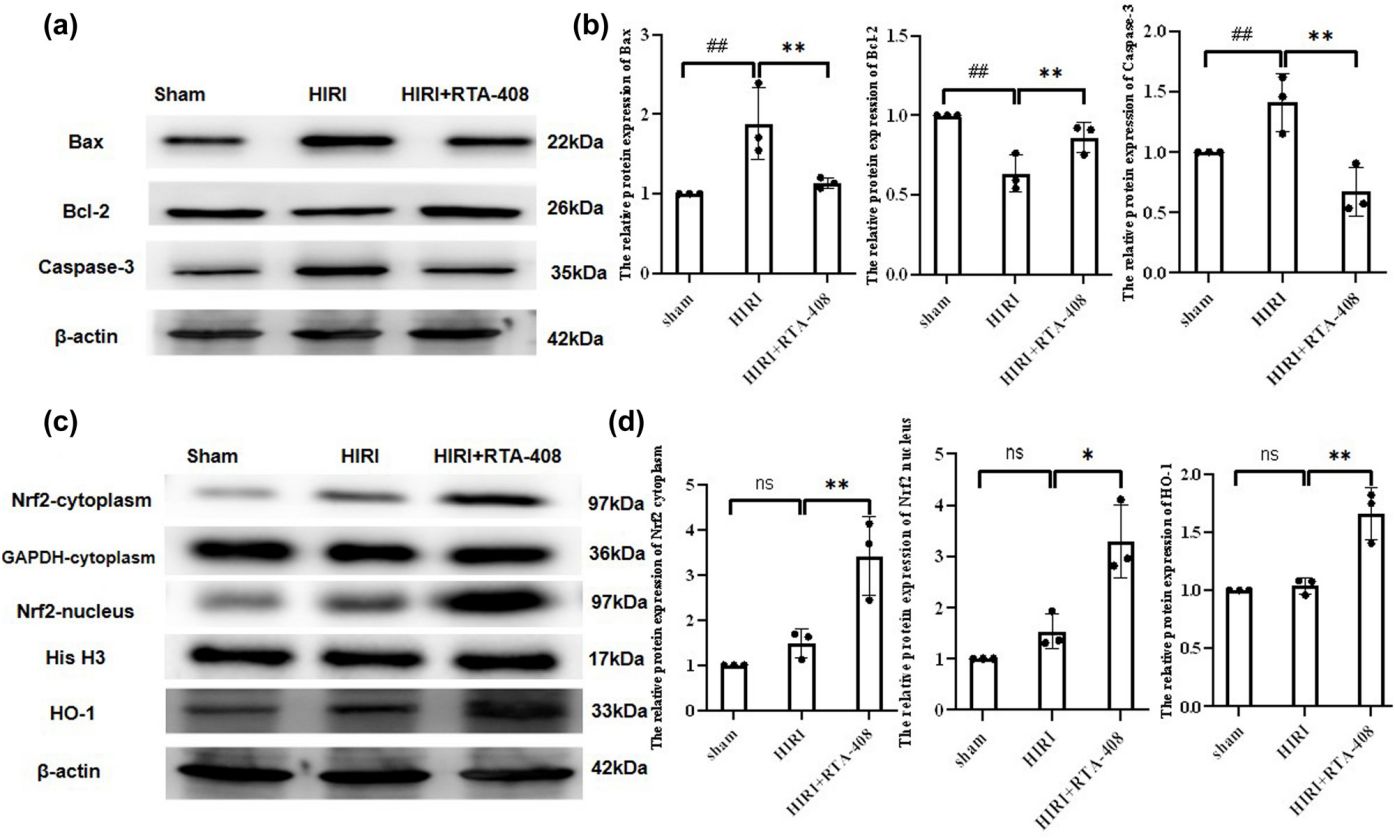
RTA-408 reduces the HIRI-induced cell apoptosis in mice liver tissues. (a) and (b) Bax, Bcl-2, and cleaved caspase-3 protein levels in mice liver samples were examined using western blot. RTA-408 activated the Nrf2/HO-1 pathway *in vivo*. (c) and (d) The protein levels of Nrf2 and HO-1 in mice hepatic tissues were assessed by western blot, *n* = 3. ^##^ *P* < 0.01 vs Sham group, **P < 0.01 and *P < 0.05 vs HIRI group.

Whether RTA-408 affected HIRI-induced cell death in mice hepatic tissues was further investigated. As revealed by TUNEL staining, the percentage of TUNEL-positive cells showed an increase in the HIRI group, which was significantly reversed by the administration of RTA-408, suggesting that RTA-408 protected liver cells from apoptosis following HIRI *in vivo* (Figure 2e and f). Then, we evaluated the protein expression of apoptosis-related proteins in mice liver tissues using western blot. Protein levels of Bax and cleaved caspase-3 increased in the mice hepatic tissues of the HIRI group and Bcl-2 reduced in the HIRI group were rescued by the treatment of RTA-408 (Figure 3a and b). Overall, RTA-408 suppresses the HIRI-induced cell death in mice liver tissues.

### 3.5 RTA-408 activated the Nrf2/HO-1 pathway in HIRI mice

We then delved into the potential mechanism of RTA-408 in the process of HIRI, and its effects on the Nrf2/HO-1 pathway was investigated. The administration of RTA-408 was demonstrated to increase the Nrf2 and HO-1 levels in mice liver tissues (Figure 3c and d).

### 3.6 Effects of RTA-408 on HIRI in *Nrf2−/−* mice

**Figure 4:**
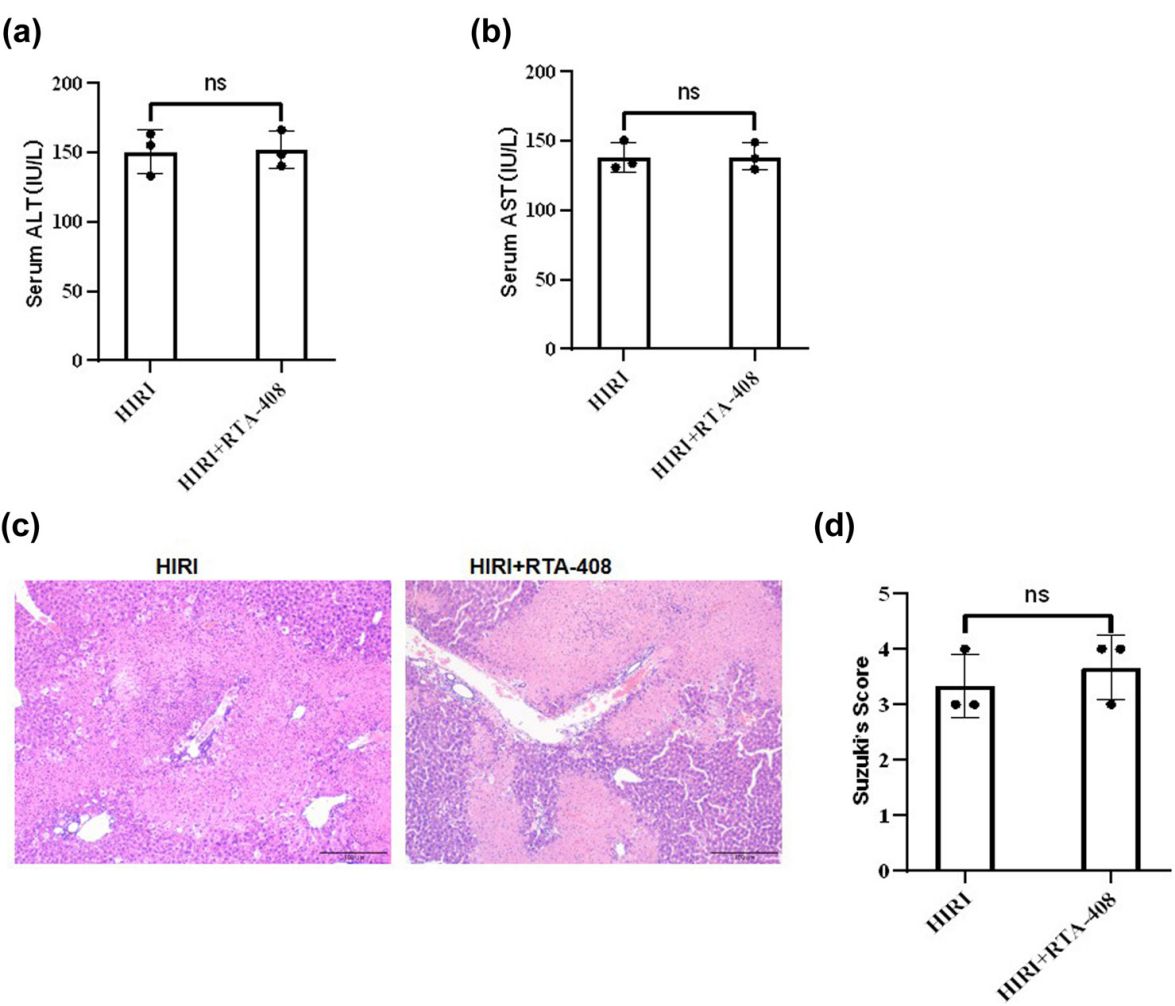
The effects of RTA-408 on HIRI in *Nrf2−/−* mice. Serum (a) ALT and (b) AST levels in *Nrf2−/−* mice subjected to indicated treatment. (c) and (d) H&E staining and Suzuki’s score for the morphology examination of mice liver samples, *n* = 3, (×200).

Whether RTA-408 protected ischemia-reperfusion injury by activating the Nrf2 signaling was further explored. *Nrf2* knockout (*Nrf2−/−*) mice were used to establish HIRI mice models. The administration of RTA-408 showed no significant effects on ALT and AST levels in mice serum relative to the HIRI group (Figure 4a and b). Moreover, H&E staining indicated that the HIRI-induced liver tissue injury was not changed by the RTA-408 treatment in comparison with the HIRI group, and histological score in the two groups showed no significant difference (Figure 4c and d). Overall, RTA-408 protected mice from liver ischemia-reperfusion injury via Nrf2 activation.

## 4 Discussion

HIRI is a common complication and major mechanism of injury following liver transplantation and hepatectomy, which has significantly affected the prognosis of patients [20–22]. The activation of Nrf2 is reported to act as a protective endogenous gene in ischemic injury, with protective effects against oxidative stress damage and HIRI [23,24]. In this study, the impact of RTA-408, a potent Nrf2 activator, on HIRI was explored using the HIRI mice models. As discovered, the administration of RTA-408 significantly improved the liver function and alleviated HIRI, oxidative stress response, inflammation as well as cell apoptosis in mice liver tissues via activating Nrf2.

Oxidative stress is essentially implicated in the regulation of HIRI [3,25]. It can induce apoptotic and necrotic cell death and promote the secretion of proinflammatory factors [23]. Substantial evidence has revealed that Nrf2 activation attenuates oxidative stress in various ischemia-reperfusion injuries [14,26,27]. Therefore, it may be useful for the targeted therapy of ischemia-reperfusion injury [28]. According to our previous study, RTA-408 alleviates oxidative stress and improves kidney function in mice with renal ischemia-reperfusion injury [11]. Liu et al. reported that RTA-408 alleviated oxidative stress damage in retinal pigment epithelial cells by activating the Nrf2 signaling [10]. In our present work, the oxidative stress markers showed significant difference between the HIRI and sham groups. Besides, the HIRI-induced increase in MDA content and reduction in SOD activity were reversed by the administration of RTA-408. Consistently, the elevated expression of inflammatory factors induced by HIRI was restored after RTA-408 treatment. Also, the HIRI-induced apoptosis was reduced by exposure to RTA-408.

Nrf2 is a transcriptional regulator of intracellular antioxidant proteins and is crucially implicated in the redox homeostasis [29]. It can bind to Keap1 in the cytoplasm under the inactivated condition and translocate to the nucleus in response to pathological stimuli such as oxidative stress [30]. HO-1, a phase II detoxifying enzyme, is up-regulated by Nrf2 [31]. It is also highly inducible by various discriminating stimuli that cause hepatic oxidative stress [11]. Both of them serve as the powerful free radical scavengers in the body [32]. In our study, RTA-408 could activate the Nrf2/HO-1 signaling in response to oxidative stress induced by HIRI in mice. Furthermore, we found that RTA-408 did not significantly influence the liver function or levels of oxidative biomarkers in the *Nrf2−/−* mice, demonstrating that RTA-408 protected from HIRI by activating the Nrf2/HO-1 signaling.

In conclusion, RTA-408 alleviates HIRI by reducing oxidative stress damage and inflammatory responses, and improving liver function in the HIRI mice models. The results of this study can provide evidence for the application of RTA-408 in the treatment of HIRI.
